# Supplementary material for: Divergences on expected pneumonia cases during the COVID-19 epidemic in Catalonia: a time-series analysis of primary care electronic health records covering about 6 million people
Source: BMC Infect Dis. 2021 Mar 20;21:283. doi: 10.1186/s12879-021-05985-0 (PMC7979451; doi:10.1186/s12879-021-05985-0)
Supplement: Supplementary file 1 — Additional file 1: Supplementary Table 1. Pneumonia ICD-10 codes. [file 12879_2021_5985_MOESM1_ESM.docx]

## Title Page

**Title:** Divergences on expected pneumonia cases during the COVID-19 epidemic in Catalonia: a time-series analysis of primary care electronic health records covering about 6 million people.

**Authors:** Ermengol Coma* ^1^, Leonardo Méndez-Boo^1^, Núria Mora^1^, Carolina Guiriguet^1,2^, Mència Benítez^1,2,^ Francesc Fina^1^, Mireia Fàbregas^1^, Elisabet Balló^1,3^, Francisa Ramos^1^, Manuel Medina^1^, Josep M. Argimon^4^

**Affiliations**

1. Sistemes d’Informació dels Serveis d’Atenció Primària (SISAP), Institut Català de la Salut (ICS), Barcelona, Spain
2. Equip d’Atenció Primària de Gòtic, Institut Català de la Salut, Barcelona, Spain
3. Equip d’Atenció Primària de Salt, Institut Català de la Salut, Girona, Spain
4. Institut Català de la Salut (ICS), Barcelona, Spain

**Supplementary Table 1: Pneumonia ICD-10 codes**

| PS_COD | PS_DES |
| --- | --- |
| J11.00 | Influenza due to unidentified influenza virus with unspecified type of pneumonia |
| J12.0 | Adenoviral pneumonia |
| J12.1 | Respiratory syncytial virus pneumonia |
| J12.2 | Parainfluenza virus pneumonia |
| J12.3 | Human metapneumovirus pneumonia |
| J12.81 | Pneumonia due to SARS-associated coronavirus |
| J12.89 | Other viral pneumonia |
| J12.9 | Viral pneumonia, unspecified |
| J13 | Pneumonia due to Streptococcus pneumoniae |
| J14 | Pneumonia due to Hemophilus influenzae |
| J15.1 | Pneumonia due to Pseudomonas |
| J15.211 | Pneumonia due to Methicillin susceptible Staphylococcus aureus |
| J15.29 | Pneumonia due to other staphylococcus |
| J15.4 | Pneumonia due to other streptococci |
| J15.6 | Pneumonia due to other Gram-negative bacteria |
| J15.7 | Pneumonia due to Mycoplasma pneumoniae |
| J15.8 | Pneumonia due to other specified bacteria |
| J15.9 | Unspecified bacterial pneumonia |
| J16.8 | Pneumonia due to other specified infectious organisms |
| J18.0 | Bronchopneumonia, unspecified organism |
| J18.1 | Lobar pneumonia, unspecified organism |
| J18.2 | Hypostatic pneumonia, unspecified organism |
| J18.8 | Other pneumonia, unspecified organism |
| J18.9 | Pneumonia, unspecified organism |
