# Supplementary material for: Divergences on expected pneumonia cases during the COVID-19 epidemic in Catalonia: a time-series analysis of primary care electronic health records covering about 6 million people
Source: BMC Infect Dis. 2021 Mar 20;21:283. doi: 10.1186/s12879-021-05985-0 (PMC7979451; doi:10.1186/s12879-021-05985-0)
Supplement: Supplementary file 2 — Additional file 2: Supplementary material 2. Details of the model. [file 12879_2021_5985_MOESM2_ESM.docx]

## Title Page

**Title:** Divergences on expected pneumonia cases during the COVID-19 epidemic in Catalonia: a time-series analysis of primary care electronic health records covering about 6 million people.

**Authors:** Ermengol Coma* ^1^, Leonardo Méndez-Boo^1^, Núria Mora^1^, Carolina Guiriguet^1,2^, Mència Benítez^1,2,^ Francesc Fina^1^, Mireia Fàbregas^1^, Elisabet Balló^1,3^, Francisa Ramos^1^, Manuel Medina^1^, Josep M. Argimon^4^

**Affiliations**

1. Sistemes d’Informació dels Serveis d’Atenció Primària (SISAP), Institut Català de la Salut (ICS), Barcelona, Spain
2. Equip d’Atenció Primària de Gòtic, Institut Català de la Salut, Barcelona, Spain
3. Equip d’Atenció Primària de Salt, Institut Català de la Salut, Girona, Spain
4. Institut Català de la Salut (ICS), Barcelona, Spain

## Supplementary material 2. Details of the model.

## Adjusted model specifications

Our global time serie has this pattern:

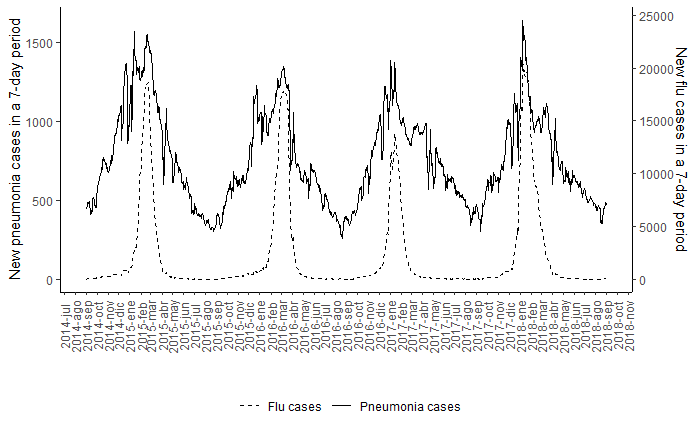


We observe a correlation between the number of pneumonia cases and the number of flu cases. We also observe a seasonal pattern. The figure of the decomposition of the time series is as follows:


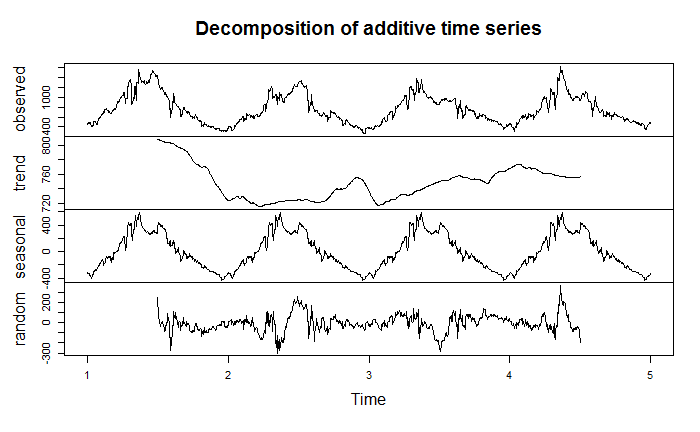


We have obtained the expected new pneumonia cases for the study period using a time series regression according to the following formulae

$$y{}_{t} =\beta_{0} + \beta_{1}\times{flu}_{t}+ \sum_{i = 2}^{365} \beta_{k}s_{tk} + e_{t}$$

where $t$is the instant time, ${flu}_{t}$ is the number of flu cases at the time $t$, $s_{ti}$is the$t$ seasonal period and $e_{t}$ is the random error.
