# Supplementary material for: Divergences on expected pneumonia cases during the COVID-19 epidemic in Catalonia: a time-series analysis of primary care electronic health records covering about 6 million people
Source: BMC Infect Dis. 2021 Mar 20;21:283. doi: 10.1186/s12879-021-05985-0 (PMC7979451; doi:10.1186/s12879-021-05985-0)
Supplement: Supplementary file 3 — Additional file 3: Supplementary material 3. Number and percentage of pneumonia diagnoses by season: total and first three months of the year. [file 12879_2021_5985_MOESM3_ESM.docx]

**Supplementary material 3. Number and percentage of pneumonia diagnoses by season: total and first three months of the year**

|  | 2014-2015 | 2015-2016 | 2016-2017 | 2017-2018 | 2018-2019 | 2019-2020 | 2020-2021 |
| --- | --- | --- | --- | --- | --- | --- | --- |
| From January to March | | | | | | | |
| Total | 16,307 (100%) | 14,196 (100%) | 12,965 (100%) | 14,135 (100%) | 15,210 (100%) | 21,290 (100%) | Not applicable |
| Younger than 15 | 5,476 (33,58%) | 4,590 (32,33%) | 3,738 (28,83%) | 3,603 (25,49%) | 4,343 (28,55%) | 3,908 (18,36%) | Not applicable |
| Between 15 and 64 | 5,634 (34,55%) | 5,150 (36,28%) | 4,391 (33,87%) | 5,176 (36,62%) | 5,845 (38,43%) | 9,598 (45,08%) | Not applicable |
| Older than 64 | 5,197 (31,87%) | 4,456 (31,39%) | 4,836 (37,3%) | 5,356 (37,89%) | 5,022 (33,02%) | 7,703 (36,18%) | Not applicable |
| Total pneumonia of the season | | | | | | | |
| Total | 42,171 (100%) | 37,883 (100%) | 39,149 (100%) | 39,447 (100%) | 42,473 (100%) | 50,039 (100%) | 9,748 (100%) |
| Younger than 15 | 15,087 (35.78%) | 13,083 (34.54%) | 12,848 (32.82%) | 11,399 (28.9%) | 13,217 (31.12%) | 8,656 (17.3%) | 428 (4.39%) |
| Between 15 and 64 | 14,219 (33.72%) | 12,736 (33.62%) | 13,094 (33.45%) | 14,032 (35.57%) | 15,373 (36.19%) | 22,416 (44.8%) | 4,917 (50.44%) |
| Older than 64 | 12,865 (30.51%) | 12,064 (31.85%) | 13,206 (33.73%) | 14,016 (35.53%) | 13,883 (32.69%) | 18,728 (37.43%) | 4,313 (44.24%) |
